# Safety and Efficacy of Renal Denervation for the Treatment of Resistant Hypertension in Patients with Chronic Kidney Disease: A Narrative Review of the Literature

**DOI:** 10.3390/biomedicines13081951

**Published:** 2025-08-09

**Authors:** Panagiotis Iliakis, Kyriakos Dimitriadis, Fotis Tatakis, Angeliki Vakka, Athanasios Sakalidis, Nikolaos Pyrpyris, Christos Fragoulis, Dimitrios Konstantinidis, Konstantinos Aznaouridis, Konstantinos Tsioufis

**Affiliations:** First Department of Cardiology, School of Medicine, National and Kapodistrian University of Athens, Hippokration General Hospital, 115 27 Athens, Greece; dimitriadiskyr@yahoo.gr (K.D.); fotistatakis@yahoo.gr (F.T.); aggvakka@outlook.com (A.V.); asakalidis@gmail.com (A.S.); npyrpyris@gmail.com (N.P.); christosfragoulis@yahoo.com (C.F.); kon_dimitris@hotmail.com (D.K.); conazna@yahoo.com (K.A.); ktsioufis@gmail.com (K.T.)

**Keywords:** arterial hypertension, chronic kidney disease, renal denervation, sympathetic nervous system, end-stage renal disease

## Abstract

Arterial hypertension is highly prevalent among individuals with chronic kidney disease (CKD), exhibiting a bidirectional association and playing a critical role in the progression of renal dysfunction. CKD affects approximately 10–12% of the global population and is often a common comorbidity in patients with true resistant hypertension. The sympathetic nervous system (SNS) plays a key role in the pathophysiological cascade of CKD-mediated hypertension. Current pharmacological therapies do not directly target SNS overactivity, highlighting the need for alternative approaches. Renal denervation (RDN), an interventional procedure that modulates both afferent and efferent renal nerve signaling, has emerged as a promising strategy for resistant hypertension with multiple pleiotropic benefits. Both preclinical and clinical trial data indicate that RDN is safe, with no significant deterioration of renal function reported in both early-stage CKD and end-stage renal disease (ESRD), as well as effective in reducing both office and ambulatory blood pressure in most studies. This review examines the pathophysiological basis for SNS overactivity in CKD, summarizes preclinical and clinical data on the safety and efficacy of RDN in this population, and discusses ongoing and future trials that may further clarify the role of RDN in CKD management and its long-term impact on renal and cardiovascular outcomes.

## 1. Introduction

Arterial hypertension is highly prevalent among individuals with chronic kidney disease (CKD), exhibiting a bidirectional association and playing a critical role in the progression of renal dysfunction [1]. CKD affects approximately 10–12% of the global population, with higher prevalence in the co-presence of hypertension diabetes, obesity, or advanced age [2]. Due to the decrease of all-cause mortality in developed countries, the incidence of CKD is growing and its early-stage recognition is of great importance [3]. Chronic kidney disease is strongly associated with resistant hypertension [4]. In the latest 2023 ESH guidelines for the treatment of hypertension, hypertension is defined as truly resistant when systolic blood pressure (BP) is ≥140 mmHg or diastolic BP is ≥90 mmHg at maximum recommended and tolerated doses of a three-drug combination, including a renin–angiotensin–system (RAS) blocker, a calcium channel blocker (CCB), and a thiazide/thiazide-like diuretic, and inadequate BP control has been confirmed by ambulatory measurements, and causes of pseudo-resistant hypertension and secondary hypertension have been excluded [5]. This is also the definition used in this manuscript and in the clinical trials further described.

The basic principles of the therapeutical management of hypertension in patients with CKD include non-pharmaceutical and pharmaceutical means of treatment [5,6]. On the basis of SNS hyperactivity in CKD establishing a self-perpetuating cycle of hypertension and renal damage, interventional means of treatment such as renal denervation (RDN) could disrupt this cycle by modulating both efferent and afferent renal signaling, offering a promising targeted strategy for resistant hypertension where conventional pharmacotherapy is insufficient. Evidence supports that RDN is not only an established treatment in resistant hypertension, as recommended in the latest ESH guidelines, but that pleiotropic effects are evident, improving glucose control, metabolism equilibrium, microvascular dysfunction and ameliorating sympathetic nervous overdrive in the failing heart [7,8].

RDN is a minimally invasive, catheter-based procedure, typically performed by a trained interventional cardiologist, aiming to disrupt the sympathetic overdrive in the sympathetic nervous system along the renal arteries [9,10]. The RDN catheter is advanced to the renal arteries via the common femoral artery, under fluoroscopic imaging guidance. Once positioned inside a renal artery, the catheter delivers energy—(RF) energy or ultrasound—to the vessel walls where the sympathetic nerves reside. Most of the nerves are located at distances of <6 mm from the arterial lumen. Ablation typically involves multiple targeted applications circumferentially and longitudinally along each renal artery to ensure adequate denervation [10]. Post-procedure, successful hemostasis with closure devices is advisable to shorten hospitalization duration. The amount of intravenous (IV) contrast agent commonly used during a renal denervation procedure is approximately 90 to 100 milliliters on average [11].

This review aims to delve into the pathophysiology of sympathetic nervous system overdrive in hypertension among individuals with CKD, present preclinical and clinical observational data regarding safety and efficacy of RDN in this population, as well as highlight ongoing and future clinical trials that could further clarify the role of RDN in CKD management and its long-term impact on renal and cardiovascular outcomes.

## 2. Materials and Methods

A literature review was performed by searching the PubMed database for studies published in the English language up to May 2025. The following key words and their abbreviations were used: “arterial hypertension” AND “chronic kidney disease” OR “end-stage renal disease” AND “renal denervation” OR “sympathetic nervous system”. Clinical guidelines, meta-analyses, systematic reviews, retrospective and prospective studies, narrative reviews, and case reports were included. Non-English-language articles and articles with unavailable full text were excluded from further analysis. The articles were considered eligible regarding animal or human data on the safety and efficacy of renal denervation in the treatment of arterial hypertension in the presence of chronic kidney disease.

## 3. Pathophysiology

CKD is increasingly recognized as a significant public health burden, closely linked with substantial cardiovascular morbidity and mortality. Its prevalence continues to rise due to improved survival rates among high-risk populations, notably those with hypertension [12,13]. Hypertension in CKD is multifactorial, with heightened sympathetic nervous system (SNS) activity playing a pivotal role. Enhanced SNS activity correlates directly with impaired renal function and perpetuates elevated blood pressure (BP), complicating therapeutic management [14,15]. Sympathetic hyperactivity in CKD initiates and sustains hypertension via multiple pathways, including increased renin secretion, sodium retention, reduced renal blood flow, and systemic vasoconstriction [6,16]. Concurrently, the renin–angiotensin–aldosterone system (RAAS) exacerbates these maladaptive responses, further promoting renal damage and systemic vascular resistance, thus establishing a vicious cycle of renal deterioration and worsening hypertension [17]. RAAS interacts with the SNS to amplify vasoconstriction, renal fibrosis, and systemic inflammation [18]. Moreover, sodium accumulation in interstitial tissue can trigger macrophage infiltration and endothelial glycocalyx dysfunction, promoting vascular inflammation and local activation of angiotensin II and aldosterone signaling pathways [19]. These inflammatory cascades exacerbate hypertension and renal injury.

Renal sympathetic nerves play a critical role in kidney physiology and pathophysiology, with specific nerve fibers regulating renal hemodynamics, renin secretion, sodium reabsorption, and overall BP control [20]. Renal efferent nerves reduce renal blood flow, increase tubular sodium reabsorption, and stimulate renin release, collectively contributing to BP elevation [21]. Afferent renal nerves also stimulate central SNS outflow, promoting systemic vasoconstriction and neurogenic hypertension [22]. Another key mechanism in CKD-associated hypertension is impaired natriuresis. As glomerular filtration rate (GFR) declines, sodium filtration is reduced, while distal tubular sodium reabsorption increases in remnant nephrons, culminating in extracellular volume expansion [23]. This sodium retention is a central driver of resistant hypertension in CKD, particularly in salt-sensitive individuals. Risk factors such as older age, Black ethnicity, diabetes, obesity, and male sex are all associated with salt sensitivity and impaired sodium excretion, leading to volume overload and elevated BP in advanced CKD [24,25,26]. These factors contribute to a higher prevalence of treatment-resistant hypertension due to underlying defects in natriuresis. Plasma noradrenaline levels, reflecting sympathetic activity, have been shown to predict mortality and cardiovascular events in patients with end-stage renal disease [27]. Furthermore, bilateral nephrectomy in CKD patients results in marked reductions in sympathetic nerve activity, arterial pressure, and vascular resistance, supporting the kidneys’ role in mediating SNS overactivity [18]. Additional support comes from renal transplant studies: patients retaining native kidneys exhibited heightened SNS activity, while those with bilateral nephrectomy had significantly lower muscle sympathetic nerve activity (MSNA) and calf vascular resistance [28].

Patients with CKD exhibit significantly elevated sympathetic nerve activity, inversely correlated with glomerular filtration rate (GFR) [29]. This elevated sympathetic tone, combined with reduced renalase activity resulting in increased catecholamine exposure, contributes directly to adverse cardiovascular outcomes and renal damage in CKD [30]. Reduced renalase activity in CKD leads to excessive catecholamine accumulation, exacerbating hemodynamic stress and sympathetic toxicity [31]. Additionally, elevated asymmetric dimethylarginine (ADMA) levels—a known endogenous inhibitor of nitric oxide synthase—are observed in CKD and end-stage kidney disease, contributing to endothelial dysfunction and vasoconstriction [32]. Disturbances in calcium-phosphate metabolism, regulation of fibroblast growth factor-23 signaling by klotho, and chronic inflammation can result in vascular calcification, increased arterial stiffness, and widened pulse pressure, especially in advanced CKD and dialysis patients [33].

Regarding ESRD, anatomical evidence supports increased nerve density in the renal artery adventitia in patients undergoing hemodialysis, correlating with heightened SNS activity compared to individuals with less severe CKD or normal renal function [34]. Afferent signaling from diseased kidneys significantly contributes to central SNS activation, as bilateral nephrectomy in ESRD patients reduces central SNS activity, systemic arterial pressure, and peripheral vascular resistance, highlighting the critical role of afferent renal signaling in systemic sympathetic overactivity [18]. The bidirectional kidney–brain axis, involving both efferent and afferent nerve pathways, mediates the vicious cycle between increased SNS activity, CKD progression, and hypertension, further reinforcing the pathophysiological basis for renal sympathetic denervation [35]. Current therapeutic strategies for CKD management, including sodium-glucose cotransporter 2 inhibitors, glucagon-like peptide-1 receptor agonists, and non-steroidal mineralocorticoid receptor antagonists, do not target SNS activity [36]. Thus, RDN uniquely addresses a crucial pathogenic mechanism in CKD. Finally, biomarker-based studies support the renoprotective neutrality of RDN. For instance, urinary CKD273 peptide signatures—predictive of CKD progression—remain unchanged up to 24 months post-RDN, suggesting no adverse molecular effect in early-stage CKD patients [37].

## 4. Sympathetic Nervous System Activity in CKD

Assessment of SNS activity is a major field in cardiovascular research, as the sympathetic overdrive plays a key role not only in hypertension but in the whole spectrum of cardiovascular disease [38]. The most common modalities for evaluating SNS activity are plasma noradrenaline (NA) levels, resting heart rate (RHR), heart rate variability (HRV) and MSNA.

### 4.1. Plasma Noradrenaline (NA) Levels

Plasma noradrenaline levels have been considered as an indicator of sympathetic neural activity since 1950s [39]. Patients with CKD have higher plasma catecholamine concentrations compared with healthy subjects [27,40], while plasma NA levels are inversely related to eGFR values [41]. Patients with end-stage renal disease and plasma NA > 75th percentile have 1.92 times higher adjusted relative risk for cardiovascular complications compared with those below this threshold [27]. However, impaired renal function may affect NA levels, as it alters the re-uptake and metabolism of NA [42].

### 4.2. Resting Heart Rate (RHR)

RHR is also a tool for assessing the status of the autonomic nervous system [43]. RHR above 90 bpm has been significantly correlated with an increased prevalence of CKD, regardless of age, BMI, and prevalence of diabetes and hypertension [44]. Heart rate above 80 bpm may be an indicator of sympathetic overactivation in patients with CKD, as values of clinic or 24 h heart rate above 80 bpm have been correlated with increased MSNA and levels of plasma NA [41]. A higher RHR, even within the normal range of below 100/min, has been associated with increased mortality and cardiovascular events in patients with CKD [43,45].

### 4.3. Heart Rate Variability (HRV)

HRV refers to the fluctuations in interbeat interval length. Heart rate is defined as the number of heart beats per minute, while HRV refers to the variation among heartbeats in a specific period of time [46]. HRV can be measured by time domain, statistical, geometric, and frequency domain methods [47]. In the time domain method, the QRS complexes of a continuous electrocardiographic record are detected and the intervals between adjacent QRS complexes (which are called normal-to-normal intervals) are calculated. In this way, variables, such as normal-to-normal interval and differences among them are calculated [48]. In the frequency domain method, power spectral density analysis quantifies the distribution of HRV power as a function of frequency [48]. Reduced HRV is considered as an independent risk factor for mortality and cardiac death in patients with cardiovascular disease and healthy populations. Patients with CKD stage 4 have decreased both time- and frequency-domain HRV measures compared with healthy individuals [49]. Among patients with CKD stage 3–5, lower HRV measures are associated with older age, presence of diabetes, higher urine albumin/creatinine ratio (ACR), and eGFR < 15 mL/min/1.73 m^2^, while higher HRV is significantly associated with a lower risk of CVD [50]. HRV also decreases along with CKD severity [51].

### 4.4. Muscle Sympathetic Nerve Activity (MSNA)

Microneurography is an accurate and sensitive method of quantifying sympathetic nerve activity in the muscles or the skin [42]. Assessing MSNA involves capturing bursts of spontaneous MSNA, in order to directly record the activity of post-ganglionic sympathetic nerve fibers that innervate vascular smooth muscle in skeletal muscle [52]. During this technique, the tip of a tungsten microelectrode is inserted into the common peroneal or median nerve [53]. Patients with CKD have higher values of MSNA compared with healthy individuals [53]. MSNA has been significantly and inversely associated with eGFR, meaning that sympathetic overactivity is also present in the initial phases of CKD [54]. Elevated MSNA has been also associated with a composite of all-cause mortality and nonfatal cardiovascular events in patients with CKD [55]. MSNA significantly decreases after RDN, with both multi-unit and single-unit MSNA demonstrating reductions over time [56,57]. These findings reinforce the therapeutic rationale of RDN in modulating both afferent and efferent renal signaling, ultimately attenuating systemic SNS activity and its pathophysiological consequences.

## 5. Preclinical Data

### 5.1. Preclinical Data in Blood Pressure Reduction

Studies in animals with CKD have shown that renal denervation is a safe procedure that is beneficial for decreasing blood pressure and improving renal function in this population. Specifically, rats with renal disease due to 5/6 nephrectomy that were subjected to dorsal rhizotomy had lower blood pressure, levels of serum creatinine, and glomerulosclerosis compared with rats with sham rhizotomy 6 weeks after the surgery [58]. Furthermore, RDN (by surrounding the renal artery with a solution of phenol) decreased blood pressure, restored the serum BUN and creatinine levels, attenuated glomerulosclerosis and tubular injury, ameliorated left ventricular hypertrophy (LVH), and normalized gamma-aminobutyric acid (GABA) levels in the nucleus tractus solitarii in CKD rats after 8 weeks of RDN [59]. Moreover, 3 weeks after selective afferent RDN (by painting the renal artery with a solution of capsaicin), rats with CKD had lower blood pressure, lower serum creatinine, decreased levels of urine protein, decreased renal and splanchnic sympathetic nerve activity (SNA), reduced renal fibrosis by 45%, reduced in-renal infiltration of macrophages, as well as lower renal hydrogen peroxide levels and NADPH oxidase activity compared with CKD rats without RDN [60]. Total RDN (by painting the renal artery with a solution of phenol) also reduced mean arterial pressure in CKD rats, while it normalized lumbar SNA, decreased splanchnic SNA, reduced plasma creatinine, and decreased proteinuria by 61% after 3 weeks of total RDN, although it cannot reverse completely tubule interstitial fibrosis [61].

Apart from rats, hypertensive sheep with CKD undergoing RDN with the Symplicity Flex catheter have also shown a significant decrease in MAP of about 10–12 mmHg, which is sustained until 30 months after RDN. Moreover, in the same group of sheep, GFR increased by 26% compared with their GFR levels before RDN at 30 months after RDN. Regarding urinary albumin levels, they had lower levels (56–60%) compared with the intact group of sheep with CKD. Sheep with CKD undergoing RDN also had lower renal vascular resistance (RVR) of ≈22–28% compared with the intact group at 30 months [62]. RDN may also improve nitric oxide (NO) bioavailability in animals with CKD, since the excretion of nitrate and nitrite in urine was ≈50–70% greater in sheep with CKD undergoing RDN at 2 and 30 months after RDN compared with the intact group. They also had elevated expression of endothelial NO synthase protein compared with the intact group [63].

### 5.2. Preclinical Data in Left Ventricular Hypertrophy and Atrial Fibrillation Susceptibility

RDN has been associated not only with amelioration of LVH, but also with a decrease of ventricular arrhythmogenicity and atrial fibrillation (AF) susceptibility in animals with CKD [64,65,66]. Specifically, dogs that undergo subtotal nephrectomy and RDN have shown significantly lower blood pressure, attenuation of LVH and CKD-induced ECG changes, higher ventricular fibrillation threshold, and lower levels of serum noradrenaline, C-reactive protein and interleukin-6 compared with CKD dogs without RDN 6 weeks after the surgical procedure [64]. Moreover, RDN decreased ventricular fibrosis and ventricular arrhythmogenicity, without deterioration of renal function, in rabbits with CKD undergoing RDN compared with the CKD group [65]. RDN also reduced left atrial (LA) diameter, LA interstitial fibrosis, AF inducibility, and the duration of AF episodes in rats with CKD undergoing RDN compared with the intact group after 16 weeks of RDN [66].

### 5.3. Preclinical Data in Cardiorenal Syndrome

RDN may also have beneficial effects on cardiorenal syndrome, which may be blood pressure-independent [67,68]. In a study of Eriguchi et al. [67], administration of N(ω)-nitro-L-arginine methyl ester (L-NAME), which is a nitric oxide synthase inhibitor, caused cardiac and renal damage in rats. Rats with cardiorenal syndrome undergoing bilateral RDN had a reduction in the severe hypertension caused by L-NAME and their blood pressure levels were comparable to those of rats with cardiorenal syndrome treated with hydralazine at week 10. Although blood pressure levels were the same in RDN and hydralazine groups, rats undergoing bilateral RDN had lower plasma and urinary norepinephrine levels, lower plasma renin activity and plasma angiotensin II levels, and suppressed proteinuria, glomerulosclerosis, and interstitial fibrosis. Also, only the RDN group had decreased AGT and angiotensin II protein levels in the left ventricle and kidneys, thus RDN ameliorated cardiac and renal RAS in rats with cardiorenal syndrome. Another study by Peleli et al. [68] compared rats that were uninephrectomized (UNX) and fed with a high salt (HS) diet and underwent RDN (UNX + HS + RDN) to rats with UNX + HS without RDN. RDN resulted in attenuation of blood pressure and cardiac hypertrophy, improvement of eGFR, and reduction of albuminuria in UNX + HS + RDN compared with UNX + HS rats, while the expression and activity of NADPH and xanthine oxidase were also lower in the UNX + HS + RDN group [68].

Furthermore, RDN may decrease acute and chronic kidney injury caused by renal ischemic reperfusion injury (IRI) in rats [69]. Rats with IRI undergoing RDN have decreased tubular lesions, fewer tunnel-positive cells and lower values of BUN at 24 h after IRI, while they also have decreased renal fibrosis and increased capillary density at 2 weeks after IRI compared with IRI rats without RDN. These changes may occur due to a modulation of miRNA expression, since RDN results in VEGF upregulation and Smad2 downregulation at 24 h after IRI, as well as in downregulation of IL-6 and elevation of Bcl-2/Bax ratio at 2 weeks after IRI. Thus, RDN may play a role in angiogenic, anti-fibrotic, and anti-apoptotic pathways [69].

### 5.4. Preclinical Data of RDN on Top of Drug Treatment

Moreover, the addition of RDN to drug treatment has been proven safe in mice with CKD [70]. Specifically, the addition of RDN to olmesartan led to a greater reduction of SBP from 14 weeks to 22 weeks of age compared with olmesartan alone in mice with CKD due to 5/6 nephrectomy, while it did not affect renal function. Heart weight per body weight and 24 h urinary norepinephrine levels were decreased in both CKD mice treated with RDN + olmesartan and CKD mice treated with olmesartan alone compared with CKD mice that were treated orally [70].

### 5.5. Preclinical Data in Re-Innervation

Regarding reinnervation, Singh et al. [62] reported a partial regrow and partial restoration of function of renal nerves at 30 months after RDN, assessed by a partial restoration of tyrosine hydroxylase (TH), calcitonin gene-related peptide (CGRP), and norepinephrine (NE) levels in kidneys. However, a study in normotensive swine showed that the reduction in renal cortical axon density and cortical NE levels, as well as the axonal loss caused by RDN with a radiofrequency RDN catheter, were sustained after 180 days [71].

## 6. Clinical Data

The important interplay between sympathetic overdrive and hypertension development and progress, the positive findings of animal studies, as well as the growing long-term evidence from well-structured randomized controlled trials have recently prompted updated recommendations regarding RDN in the European guidelines for the treatment of hypertension [5,72]. However, its applicability is still limited in patients with eGFR > 45 mL/min/1.73 m^2^, suggesting that it can be used only in patients with stage CKD 3a and above; there is a lack of randomized evidence regarding patients with more impaired renal function. Apart from reduced renal function, we should also take in consideration structural or anatomical abnormalities, like renal artery length < 20 mm, diameter < 4 mm, or the presence of stents or aneurysms, that are exclusion criteria for RDN [73]. Sub-analysis of the 36 months of data from the Global SYMPLICITY Registry in patients with CKD stage 3a (eGFR 46–60 mL/min/1.73 m^2^), demonstrate that after adjusting for baseline characteristics, ABP decrease did not differ among patients with or without CKD, implying its efficacy in this population [74]. In this section we provide clinical data, mainly small and observational, regarding the safety and efficacy of RDN in patients with resistant hypertension and CKD with eGFR < 45 mL/min/1.73 m^2^ and ESRD (see Table 1. Clinical trials of RDN in patients with resistant hypertension and CKD, and Table 2. Clinical trials of RDN in patients with hypertension and ESRD).

### 6.1. Moderate-to-Severe Chronic Kidney Disease

In 2012, Hering et al. were among the first to evaluate the safety and efficacy of RDN in patients with resistant HTN and moderate to severe CKD [75]. Towards this purpose, the investigators enrolled 15 patients with resistant hypertension and stage 3–4 CKD (mean eGFR being 31 mL/min/1.73 m^2^); bilateral RDN was performed and the patients were followed up for 12 months. The investigators demonstrated that the procedure was not only safe, without any procedure-related major adverse events or deterioration of renal function, but also efficient, with significant documented reduction of both office and ambulatory BP at 6 months. This trial was the first in-man evaluation of the intervention in patients with eGFR < 45 mL/min/1.73 m^2^, although limited by its small sample size and short follow-up period. Ott et al., a couple of years later, conducted an observational study, with 27 patients with CKD stages 3 and 4 and resistant hypertension, with RDN being performed using the Symplicity Flex RDN System. Compared to the aforementioned study by Hering, in this trial the patients were characterized by higher level of renal function, with a mean eGFR of 48.5 mL/min/1.73 m^2^. They were followed up for 12 months [76]. Although its efficacy in reducing BP was highly anticipated, RDN was proven to be associated with a significant improvement in kidney function progress, estimated by regression slope of eGFR change before and after RDN, specifically with an improvement of the aforementioned calculated outcome of +1.5 ± 10 (*p* = 0.009), 12 months post procedure.

Evaluating the long-term safety and efficacy of the procedure, Kiuchi et al. conducted a multi-center observation trial and enrolled 30 patients with resistant hypertension and CKD (mean eGFR of 61.9 mL/min/1.73 m^2^), that were followed up for 24 months after the procedure [77]. They documented not only significant reduction in both OBP and ABPM at follow-up, but also a significant decrease of the average number of antihypertensive drugs (3.2 ± 1.3 at follow-up, compared to 4.6 ± 1.3 at baseline, *p* < 0.0001). Office BP was decreased from 185 ± 18/107 ± 13 to 131 ± 15/87 ± 9 mmHg and ambulatory BP was decreased from 152 ± 17/93 ± 11 to 132 ± 14/84 ± 12 mmHg, *p* < 0.0001 for both comparisons. Regarding renal function, there was no statistically significant difference in eGFR at follow-up, compared to baseline. Although observational in design and with a small sample size, this study highlighted the long-term impact of RDN in patients with CKD, accompanied by safety and no deterioration of renal function; however, the mean eGFR in this trial was higher compared to other studies, implicating a better kidney environment. During the same period, Hering et al. carried out a similar observational trial, with longer follow-up than the standard time period of 12 months, in which they enrolled 46 patients with resistant HTN and mean eGFR of 46.2 mL/min/1.73 m^2^, undergoing RDN and being followed up for 24 months [78]. Compared to Kiuchi et al.’s study [77], in this trial the patients were characterized from lower mean eGFR [78]. There were no procedure-related adverse events. It is noteworthy that RDN was associated with improvement of renal function evaluated by eGFR at 3 months (+3.73 ± 1.64 mL/min/1.73 m^2^ compared to baseline, *p* = 0.02), with no significant changes, compared to baseline, at 6, 12, and 24 months post-procedure. It is noteworthy that the investigators had evaluated the decline in renal function from 60 months prior to the procedure up to the time frame of the procedure, demonstrating that the performance of RDN slowed down renal function decline; that alongside the fact that BP changes were unrelated to eGFR changes, this could imply potential pleiotropic effects of RDN on the kidneys.

In a smaller feasibility study of 11 enrolled patients with moderate-to-severe CKD and mean eGFR 29.3 mL/min/1.73 m^2^, Hameed et al. evaluated the utilization of carbon dioxide (CO_2_) as a sole contrast agent during the performance of RDN in an open-label setting [79]. This trial showed that CO_2_ is a safe and feasible option towards undergoing RDN in patients with resistant hypertension, accompanied by non-significant adverse events (one patient developed groin hematoma) and a trend towards improvement of both office BP and albuminuria. In Regina RDN study, Prasad et al. evaluated the efficacy of RDN regarding changes in central BP and arterial stiffness changes, evaluated by pulse-wave velocity (PWV) [80]. Towards this aim, they enrolled 25 patients, with stage 3–4 CKD and resistant hypertension, undergoing RDN and followed up for 24 months. Although significant improvement in office BP reduction was noted, there was a non-significant numerical improvement of central BP measurement and aortic stiffness; no safety concerns were raised.

SymplicityHF was an open-label clinical trial, designed to evaluate the feasibility of RDN in patients with resistant hypertension, CKD, and heart failure (HF) [81]. They enrolled 39 patients with HF with reduced ejection fraction (<40%), who had undergone RDN, and were followed up for 12 months. The investigators demonstrated that RDN was associated with significant reduction of NT-proBNP, without any significant change of renal function. Regarding safety, one patient died, one patient had a myocardial infarction and there were 12 hospitalizations for HF; it is noteworthy though to add that the study population was of higher cardiovascular risk, compared to other trials, therefore safety events were to be anticipated. A recent metanalysis of the aforementioned trials demonstrated that RDN was not only safe in patients with CKD, but also associated with a persistent and significant decrease of OBP, ABPM, and ACR at 24 months and 3–6 months post-procedure, respectively [82].

### 6.2. End-Stage Renal Disease

Exploring its full potential in severely impaired renal function, Schlaich et al. conducted yet another feasibility trial to evaluate the safety and efficacy of RDN in patients with resistant hypertension and ESRD [83]. They enrolled 12 patients that were in dialysis, and after thorough evaluation of autonomic activity measurement and renal anatomy assessment, RDN was performed in 9 patients; 3 patients were excluded due to the presence of renal artery atrophy. All 9 patients were closely followed up for 24 months. They showed that even at the basis of dialysis-dependence, RDN was safe (only one patient developed procedure-related femoral pseudo-aneurysm). A sustained (but not statistically significant) reduction of OBP was documented—the trial was not powered for efficacy—but it is also noteworthy that the mean average number of antihypertensive drugs was reduced at follow-up. A similar feasibility trial enrolled 9 CKD patients (6 hemodialysis and 3 peritoneal dialysis) with resistant HTN; bilateral RDN with the multipolar EnligHTN catheter was performed and they were followed up for 12 months [84]. Regarding safety, one patient developed procedure-related femoral pseudo-aneurysm needing vascular surgical treatment, and one patient died 3 months post-procedure, but the death was due to dialysis-related complications. Furthermore, RDN was associated not only with reduction of OBP by −24/13 mmHg, ABPM by 14% at 12 months, but also with a decrease of echocardiographically evaluated left ventricular mass by 13% at 12 months, compared to baseline.

Followingly, Ott et al. conducted a single-center prospective pilot study to evaluate the efficacy of RDN in patients with resistant hypertension and ESRD, regarding ABPM reduction [85]. Six patients with ESRD on dialysis were enrolled and followed-up for 6 months; RDN was performed successfully, without any procedure-related adverse events, and ABPM was performed at baseline and 6 months after RDN. They showed that RDN was associated with a significant decrease in mean ABPM 20/15 ± 17/12 mmHg (*p* = 0.043). Similar results were found also for daytime and nighttime measurements. It is also important to mention that RDN did not affect hemodialysis parameters. Scalise et al. designed a prospective, non-randomized study in the same population with a longer follow-up; they enrolled 12 patients with resistant hypertension and ESRD willing to undergo percutaneous RDN and 12 patients with similar baseline characteristics under medical treatment serving as control [86]. All patients were followed up for 12 months for both groups and underwent office and ambulatory BP assessment at baseline, 1-, 3-, 6-, and 12-months post-baseline. Patients undergoing RDN were associated with statistically significant reduction of office and ambulatory BP, compared to control group, while comparisons remained significant for both daytime and nighttime measurements; in addition, the BP-lowering effect of RDN proved to be stable and sustained during all study time frames.

Schneider et al. conducted a rather sophisticated randomized controlled trial, enrolling 18 patients with post-renal-transplant hypertension to either RDN and optimal medical treatment or medical treatment alone [87]. All patients underwent both office and ambulatory BP measurements at baseline and 6 months after. They demonstrated that RDN, compared to medical treatment alone, was associated with significant reduction of systolic office BP and systolic nocturnal ambulatory BP (−23.3 ± 14.5 mmHg, *p* = 0.001 and −10.38 ± 12.8 mmHg, *p* = 0.06, respectively). Although there were concerns due to the highly sensitive population enrolled, there were no adverse events documented in either group.

## 7. Ongoing Trials and Future Perspectives

The recent changes in European guidelines for the treatment of hypertension, based on the landmark trials of RDN and the long-term results of clinical trials, as well as the promising findings of smaller studies in patients with CKD, have led to the design of more structured trials evaluating the efficacy of the procedure in this phenotype [5,72]. This is even more highly supported by the pleiotropic effect of the procedure in microcirculation and metabolic dysfunction [88,89], as well as targeting symptomatic relief of flank or abdominal pain associated with renal cysts in patients with autosomal dominant polycystic kidney disease (ADPKD) [90].

The Renal Denervation in Chronic Kidney Disease—RDN-CKD Study (RDN-CKD) (NCT04264403) is a prospective, multicenter double-blind, randomized clinical trial (1:1), with an unblinded interventionalist and blinded site investigators and a sham-controlled design. It plans to enroll 80 patients diagnosed with stage 3a or 3b CKD and uncontrolled hypertension (1–5 drug classes with RAS-blocking agents being mandatory, systolic office BP ≥ 140 mmHg confirmed by 24 h ambulatory BP systolic ≥ 130 mmHg) and its primary outcome is the change, compared to baseline, systolic 24 h ABPM, after 6 months follow-up between the two study arms. REducing Sympathetic Activity Through Ultrasound-based Renal deneRvation in Excessive Cardiovascular Risk populaTions (RESURRECT) is another non-randomized clinical trial, aiming to evaluate the efficacy of RDN in reducing sympathetic overdrive using the Paradise denervation system in patients with CKD, HF, and/or ESRD (NCT05703620). Towards this aim, the study investigators plan to enroll 75 patients with either CKD stage 3a/b or ESRD on stable renal replacement therapy or mild-to-moderate HF with reduced ejection fraction, and the primary outcomes are the change since baseline of NA spillover at 3 months post-procedure and the change since baseline of MSNA at 12 months post-procedure. Regarding the whole spectrum of renal disease, Effect of Renal Denervation on Bood Pressure in Patients on Hemodialysis (RDN-HD) is a prospective, single-center feasibility study that aims to enroll 12 patients with ESRD and uncontrolled resistant hypertension (NCT06556407). The study will evaluate the safety and efficacy of ultrasound based renal denervation in this severely impaired phenotype and the primary safety outcome will be major adverse events during 6 months post-procedure and the primary efficacy outcome will be changes since baseline in both office and ambulatory BP at 6 months post-procedure. Data from both non-randomized and randomized trials in patients with ESRD at and not on hemodialysis with difficult-to-control hypertension regarding the safety and efficacy of the intervention are of great importance and are utterly anticipated, to explore more therapeutical options.

## 8. Conclusions

The current body of evidence highlights that RDN is a promising therapeutical alternative for patients with resistant hypertension across the whole spectrum of CKD severity, including ESRD. Preclinical as well as early feasibility clinical data support the safety of RDN in this phenotype, accompanied by important amelioration of invasive and non-invasive SNS activity indices and significant reduction of both office and ambulatory BP. Notably, these benefits were achieved without compromising kidney function, even in low eGFR strata. Furthermore, possible pleiotropic effects of RDN on renal function, as implied by stabilization or slowed decline of eGFR in some studies, warrant further exploration.

Although most clinical trials were small and observational, and many were not powered for efficacy endpoints, the consistent trend toward BP reduction and absence of major procedure-related complications across diverse CKD populations is reassuring. Overall, while current data support the safety and efficacy of RDN in resistant hypertension with CKD, large-scale, randomized, controlled trials with long-term follow-up are needed to conclusively establish its role, particularly regarding renal outcomes and cardiovascular risk reduction, provided the pleiotropic effects of the procedure on the whole cardiometabolic spectrum. Future studies should also aim to better define patient subgroups that may derive the greatest benefit from this intervention.

## Figures and Tables

**Table 1:** Clinical trials of RDN in patients with hypertension and CKD.

Study (Author/Year)	n	Follow-Up (Months)	Age (Mean)	eGFR (Mean)	CKD (Stage)	Safety	Efficacy
Hering/2012	15	12	61 ± 9	31.2 ± 8.9	3–4	No renal function deterioration No procedure—related events	↓ OBP −33/−19 ↓ Nighttime ABPM
Ott/2015	27	12	63.4 ± 9.4	48.5 ± 12	3–4	Improvement of eGFR regression slope: ▪ Before: −4.8 ± 3.8 per year ▪ After: +1.5 ± 10	Significant ↓ OBP 20/8 Significant ↓ ABPM 9/4
Kiuchi/2016	30	24	55 ± 10	61.9 ± 23.9	2–4	NS difference in eGFR Significant improvement of ACR	↓ OBP, ABPM ↓ average number of AHD
Hering/2017	46	24	66 ± 9	46.2 ± 13.0	3–4	Improvement of eGFR at 3 months NS changes at 6, 12 and 24 months	Significant ↓ daytime ABPM (148 ± 19 vs. 136 ± 17)
Hameed/2017	11	6	57.3 ± 4.9	29.3 ± 6.6	3–4	Groin hematoma (n = 1) Groin pain (n = 1)	NS ↓ systolic OBP −14 Significant ↑ systolic dABPM
Hopper/2017	39	12	65 ± 11	52.6 ± 15.3	3–4	1 death, 1 myocardial infarction 12 hospitalizations for HF	Sig. ↓ NT-proBNP, OGTT NS change of eGFR, LVEF
Prasad/2019	25	24	62.8 ± 12.4	37.5 ± 4.8	3–4	No deterioration of renal function	NS improvement of cBP, PWV
Liu/2023	8	6		46.5 ± 33.0	1–5	No procedure—related events No deterioration of renal function	Significant ↓ OBP −22.1/11.0 Significant ↓ ABPM −18.0/7.7

ABPM: ambulatory blood pressure, AHD: antihypertensive drugs, cBP: central blood pressure, CKD: chronic kidney disease, eGFR: estimated glomerular filtration rate, NS: non-significant, NT-proBNP: N-terminal pro b-type natriuretic peptide, OBP: office blood pressure, OGTT: oral glucose tolerance test, PWV: pulse-wave velocity, RDN: renal denervation, ↓: Reduction.

**Table 2:** Clinical trials of RDN in patients with hypertension and ESRD.

Study (Author/Year)	n	Follow-Up (Months)	Age (Mean)	eGFR (Mean)	CKD (Stage)	Safety	Efficacy
Schlaich/2013	9	24	47.4 ± 13.0	<15	Dialysis	1 patient developed femoral pseudo-aneurysm, needing vascular surgery	NS reduction of OBP ↓ of average number of AHD
Hoye/2017	9	12	59 ± 9	<15	Dialysis	1 patient: femoral pseudoaneurysm (needing vascular surgery) 1 patient: MI 4 days post-RDN 1 death from dialysis-related complications	↓ LV mass by 8% (3 months) ↓ LV mass by 13% (12 months) ↓ OBP 24/13 ↓ ABPM by 14%
Ott/2019	6	6	42.5 ± 15.2	<15	Dialysis	No procedure-related events No change in hemodialysis parameters	Significant ↓ in mean ABPM 20/15
Scalise/2020	24	12	56.5 ± 16.5	<15	Dialysis	No procedure-related events	Significant ↓ in OBP, ABPM

ABPM: ambulatory blood pressure, AHD: antihypertensive drugs, CKD: chronic kidney disease, eGFR: estimated glomerular filtration rate, ESRD: end-stage renal disease, LV: left ventricle, MI: myocardial infarction, NS: non-significant, RDN: renal denervation6.1. Moderate-to-Severe Chronic Kidney Disease, ↓: Reduction.

## Data Availability

As this is a review article, no data were created.

## Abbreviations

The following abbreviations are used in this manuscript:

ACR	Albumin/creatinine ratio
CKD	Chronic kidney disease
CO_2_	Carbon dioxide
ESRD	End-stage renal disease
eGFR	Estimated glomerular filtration rate
GFR	Glomerular filtration rate
HF	Heart failure
HRV	Heart rate variability
LVH	Left ventricular hypertrophy
MSNA	Muscle sympathetic nerve activity
NA	Noradrenaline
NT-proBNP	N-terminal pro b-type natriuretic peptide
PWV	Pulse-wave velocity
RAAS	Renin–angiotensin–aldosterone system
RHR	Resting heart rate
RDN	Renal denervation
SNA	Sympathetic nervous activity
SNS	Sympathetic nervous system
